# Identifying Barriers and Facilitators to Diet and Physical Activity Behaviour Change in Type 2 Diabetes Using a Design Probe Methodology

**DOI:** 10.3390/jpm11020072

**Published:** 2021-01-26

**Authors:** Kevin A. Cradock, Leo R. Quinlan, Francis M. Finucane, Heather L. Gainforth, Kathleen A. Martin Ginis, Ana Correia de Barros, Elizabeth B. N. Sanders, Gearóid ÓLaighin

**Affiliations:** 1 Physiology Department, School of Medicine, National University of Ireland Galway, Galway, Ireland; k.cradock1@nuigalway.ie; 2 Electrical & Electronic Engineering, School of Engineering, National University of Ireland Galway, Galway, Ireland; gearoid.olaighin@nuigalway.ie; 3 Centre for Research in Medical Devices (CÚRAM), Science Foundation of Ireland, Galway, Ireland; 4 Bariatric Medicine Service, Galway Diabetes Research Centre, Health Research Board Clinical Research Facility, Galway, Ireland; Francis.Finucane@hse.ie; 5 School of Health and Exercise Sciences, Faculty of Health and Social Development, The University of British Columbia, Kelowna, BC V1V 1V7, Canada; heather.gainforth@ubc.ca (H.L.G.); kathleen_martin.ginis@ubc.ca (K.A.M.G.); 6 Fraunhofer Portugal AICOS (Fraunhofer Center for Assistive Information and Communication Solutions), Rua Alfredo Allen, 455/461, 4200-135 Porto, Portugal; anacorreiadebarros@gmail.com; 7 Department of Design, The Ohio State University, 100 Hayes Hall, 108 North Oval Mall, Columbus, OH 43210, USA; liz@maketools.com

**Keywords:** Design Probe, Human-Centred Design, diet, physical activity, barriers, facilitators, Type 2 Diabetes, behaviour change, thematic analysis

## Abstract

Treatment of Type 2 Diabetes (T2D) typically involves pharmacological methods and adjunct behavioural modifications, focused on changing diet and physical activity (PA) behaviours. Changing diet and physical activity behaviours is complex and any behavioural intervention in T2D, to be successful, must use an appropriate suite of behaviour change techniques (BCTs). In this study, we sought to understand the perceived barriers and facilitators to diet and PA behaviour change in persons with T2D, with a view to creating artefacts to facilitate the required behaviour changes. The Design Probe was chosen as the most appropriate design research instrument to capture the required data, as it enabled participants to reflect and self-document, over an extended period of time, on their daily lived experiences and, following this reflection, to identify their barriers and facilitators to diet and PA behaviour change. Design Probes were sent to 21 participants and 13 were fully completed. A reflective thematic analysis was carried out on the data, which identified themes of food environment, mental health, work schedule, planning, social support, cravings, economic circumstances and energy associated with diet behaviour. Similar themes were identified for PA as well as themes of physical health, weather, motivation and the physical environment.

## 1. Introduction

The escalating prevalence of type 2 diabetes T2D [1] is driven primarily by unhealthy eating habits, sedentary lifestyles, urbanization, economic development and an increasingly ageing population [2]. Treatment of T2D typically involves pharmacological methods and adjunct behavioural modifications focused on changing diet and physical activity behaviours [3]. However, changing any behaviour is challenging, but changing diet and physical activity behaviours is particularly complex as these behaviours are influenced by the interplay of behavioural, environmental, emotional and social factors [4,5,6] in addition to neuroendocrine and genetic influences [7]. 

Any behavioural intervention in T2D, to be successful, must address these factors and do so through the application of an appropriate suite of behaviour change techniques (BCTs) [8]. A BCT is defined as “an observable, replicable, and irreducible component of an intervention designed to alter or redirect causal processes that regulate behaviour” [9] and recent systematic reviews have documented the BCTs associated with successful clinical outcomes in diet and/or physical activity interventions in T2D [3,10,11].

In this study, we were interested in gaining a better understanding of the lived experiences of persons with T2D and their perceived barriers and facilitators to diet and physical activity behaviour change. We are seeking this knowledge with the view to designing (using a Human-Centred Design methodology) artefacts, with an appropriate set of embedded BCTs, to support lifestyle changes in persons with T2D, specifically diet and physical activity lifestyle changes. 

Human-Centred Design (HCD) is a design philosophy that seeks to place the end user at the centre of the design process [12]. In the 1980s, Donald Norman put forward guidelines that designers could follow so that their designs would achieve good usability outcomes by adopting a HCD approach [13].

**Figure 1:**
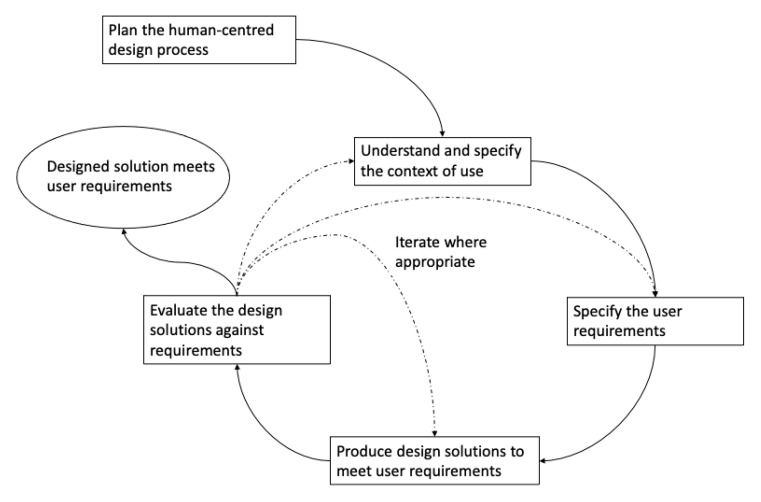
Human-Centred Design Process image based on ISO 9241-210 Ergonomics of human–system interaction—Part 210: Human-centred design for interactive systems. The solid lines represent transitions that must occur and the dotted lines are transitions that may occur depending on how the processes evolve. [12].

In HCD, the design process (Figure 1):

- requires an explicit understanding of users, tasks, and environments
- has users involved throughout the design and development process
- is driven and refined through user-centred evaluation
- is an iterative process
- addresses the whole user experience
- involves a design team with multidisciplinary skills and perspectives

In HCD, there is widespread recognition that is it important for designers to gain empathy with the users for whom they are designing and that it serves to inform and to inspire the designer to create products that better fit the needs of the user [14]. Kouprie et al. described empathy as being related to a “*deep understanding* of the user’s circumstances and experiences and which involves *relating to*, more than just *knowing about* the user” [14] (p. 440). 

Thus, the first phase of HCD is User Research, where the “lived experiences” of the target users are explored and where a deep understanding of the context of use is developed [12]. Having considered a range of design research methods [15], the Design Probe method was chosen as the most appropriate design research instrument to capture the required data, for the following reasons:

- Design Probes allows the participant to interact with them on a daily basis in their own time and space, reflecting on their lived experience and capturing that reflection by adding content to the probe each day [16].
- A Design Probe is an exploratory method that does not conform to traditional ethnographic methods but is capable of yielding rich insights [17], perhaps unattainable by other established methods.
- Design Probes can use elements or combinations of methods such as diary-writing, collage-making, photography, drawing, games and voice recording [18,19].
- Design Probe tasks can be paper-based, digital, online [20] or combinations of both [21], for longer [22] or shorter durations [23].

Mattelmäki refers to Design Probes as an approach used to understand human phenomena and explore design opportunities, where the probes involve user participation by means of self-documentation [16]. With probes, “users collect and document the material working as active participants” and with probe assignments, “users record their experiences as well as express their thoughts and ideas” [16]. Mattelmäki refers to these assignments as being a means to “record the participants’ daily lives including social environment, needs, feelings, values and attitudes” [16]. Mattelmäki refers to the process of collecting data from several situations as giving “a more reliable and credible idea of a person than recording just one situation by means such as observation” [16]. She describes the probe as “an attempt to minimise the observer’s possible influence on the person observed because the presence of the observer is likely to change the behaviour” and that “self-documentation can record context-related experiences as they occur, minimising retrospection” [16]. “Experiences are recorded in a more genuine form there and then” than might occur in “situations such as group interviews conducted afterwards” [16]. “When people look back, they do not necessarily remember the situations and sensations that they have experienced sufficiently well or their reminiscences are contaminated or distorted” [16].

A Design Probe, which enables participants with T2D to self-document (and in the process reflect on) their daily lived experience, is described. We present results from the Design Probe and comment on the experience of using this methodology to gain insight towards artefact design to support diet and physical activity behaviour change in persons with T2D.

## 2. Materials and Methods

This paper adhered to the conduct and reporting guidelines of the consolidated criteria for reporting qualitative research (COREQ) [24]. Ethical approval was obtained from the University Hospital Galway Clinical Research Ethics Committee. The ORBIT model (Obesity Related Behavioral Intervention Trials) is a framework which provides a three phase pathway for translating behavioural change findings into behavioural treatments for treatment and prevention of chronic illness [25] guided our overall approach with our earlier systematic reviews [3,11], corresponding to Phase 1a, and Design Probes aligning with Phase 1b. 

### 2.1. Recruitment/Subjects

Participants were recruited from the CROI CLANN programme at the Croí Centre, Galway [26] and the Diabetes Centre, University Hospital Galway, between August 2017 and February 2019. The CROI CLANN programme is a 10 week lifestyle intervention run by a multidisciplinary team and incorporates group exercise, nutrition and lifestyle education sessions each week [26]. The programme consists of a pre- and post-assessment, weekly exercise sessions (number of sessions for each topic in brackets) and talks on healthy eating (3), lifestyle changes (2), physical activity (1), psychological perspectives on weight control (1) and cardiovascular risk factors (1) [26]. Twenty-one participants volunteered to participate in the study and provided written informed consent. Participants were required to be diagnosed with type 2 diabetes and to have previously completed the CROI CLANN lifestyle intervention programme. 

### 2.2. Design Probe Design Philosophy 

Our approach in designing the probes was to be empathetic, non-judgmental (particularly around diet and physical activity practices), and to build rapport with the participants. The Design Probe was designed as an A5 workbook with a wide spiral binding, so that it could open flat on a table or be used on a variety of surfaces to facilitate handwriting. We sought to facilitate the deeply personal nature of self-documentation and self-reflection through handwriting as participants created an image of themselves and their lives through the written activities and reflections [16]. Colour is a powerful design tool, sometimes aggressively used in advertising, media and instructions [16,27]; therefore, we purposely used a colour scheme that was non-threatening, with soft tones using a light cream background colour, and instructions were provided in blue rather than harsher black text. 

### 2.3. Design Probe Process

**Figure 2:**
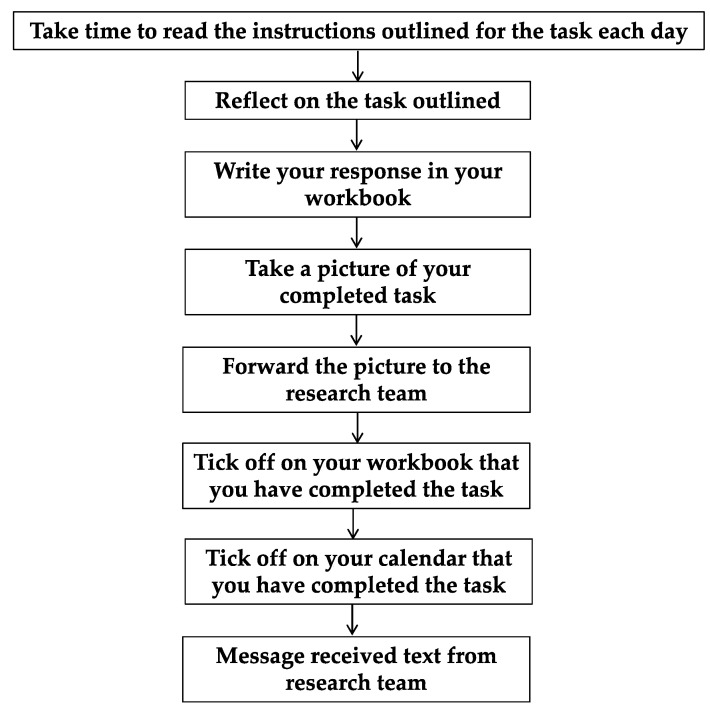
Instructions to the participant in completing Design Probe each day.

Participants were posted a folder containing pens, a calendar, fridge magnets and the Design Probe implemented as an A5 sized workbook. Instructions were incorporated into the workbook but sample answers were not, so as not to influence or frame the respondents’ answers in ways that might reduce the authenticity of responses. The Design Probe was to be used daily, with specific tasks for each day. Immediately after completing the task on the probe, participants were asked to take a photo of it and send it to the research team via text, email or a messaging App. Participants were then asked to tick the box on the bottom of the task sheet and on the fridge calendar. This helped the research team to monitor if tasks were being completed and how they were completed (Figure 2). A daily text reminder and “message received” text was sent to participants each day once they had forwarded on their completed task. Participants were telephoned or texted if they missed two consecutive days. 

### 2.4. Design Probe Design

**Figure 3:**
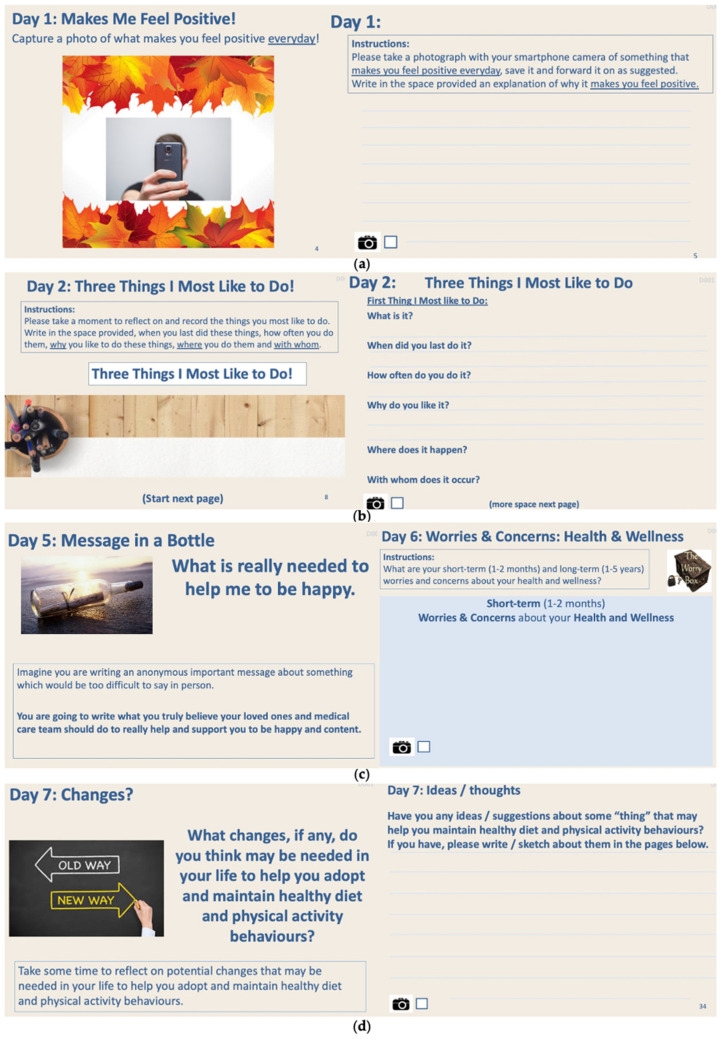
Samples of the Design Probe tasks for the participants Day 1 (**a**) Day 2 (**b**) Day 5 & 6 (**c**) and Day 7 (**d**).

**Figure 4:**
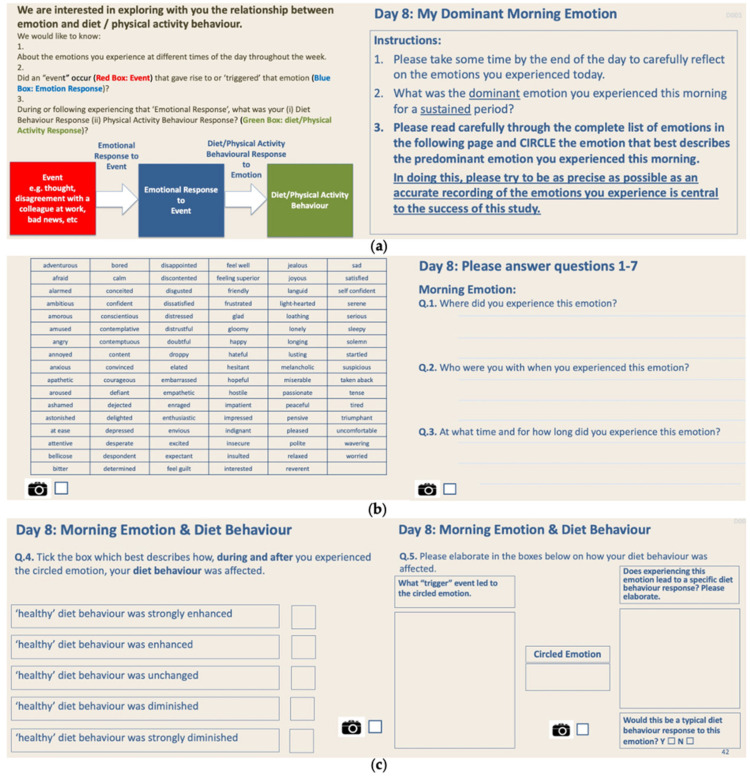
The Design Probe sought to elicit reflection and sensitisation of the participants to the inter-connection between emotions they experienced (**a**), the context in which those emotions occurred (**b**), how those emotions might have influenced their diet behaviours and what were the trigger events that may have triggered those emotions (**c**). A similar process was adopted for physical activity behaviours. This process was repeated each day for one week of reflection on the dominant morning emotion, one week of the dominant afternoon emotion and one week of the dominant evening emotion.

The Design Probe was designed using an iterative design process, where various concepts were tested with pilot users before the final version of the probe evolved. The probe contained a combination of writing and photography tasks over 31 days (Figure 3 and Figure 4 provide some samples of these tasks).

In the probe, participants were asked to complete:

- something that “makes me feel” positive and something that “makes me feel” negative in photo-based tasks (Day 1)
- three things participants most like to do and the context in which those things occur (Day 2)
- three things participants least like to do and the context in which those things occur (Day 3)
- examples of where the participants believe that they were/are not being listened to; (Day 4)
- write an anonymous message in a bottle about what is really needed to make me happy written to both loved ones and to their medical care team (Day 5)
- short-term and long-term health and wellness hopes/dreams and worries/concerns; (Day 6)
- changes needed in my life to help adopt healthy diet and physical activity behaviours (Day 7)
- ideas to maintain healthy diet and physical activity behaviours (Days 7–28)
- reflection and sensitisation of the participants to the inter-connection between emotions they experienced, the context in which those emotions occurred, how those emotions might have influenced their diet behaviours and how those emotions might have influenced their physical activity behaviours and what were the trigger events that may have triggered those emotions; this process was repeated for 7 days of dominant morning emotions, 7 days of dominant afternoon emotions and 7 days of dominant evening emotions; (Days 8–14 morning, Days 15–21 afternoon, Days 22–28 evening)
- barriers to diet behaviour change—what is it, when did it occur, where did it occur, who did it occur with (Day 29)
- barriers to physical activity behaviour change—what is it, when did it occur, where did it occur, who did it occur with (Day 29)
- facilitators to diet behaviour change—what is it, when did it occur, where did it occur, who did it occur with (Day 30)
- facilitators to physical activity behaviour change—what is it, when did it occur, where did it occur, who did it occur with (Day 30)
- best ideas from those generated over Days 7–28

In these daily tasks, particular emphasis was placed on the participants reporting on the context for the issue in question (where, when and with whom). Initial tasks in the Design Probe were easy to complete (Days 1–6), gently introducing participants to the sensitization process of self-documentation and self-reflection (Days 7–28). Day 7 asked participants about what changes participants felt were required to help them adopt and maintain healthy diet and physical activity behaviours. Days 8–28 captured emotions, using an adapted emotional framework [28], and asked the participants to report on the impact of that emotion on their diet and physical activity and to also identify the trigger event for the emotion experienced (Figure 4).

The goals of the tasks in Days 1–28 were to help the participants to become more self-aware and in particular to become more aware of how the context in which they live (what, where, who) and their emotions (and the context in which those emotions occur) influence their behaviours around diet and physical activity. This process of making the participants more self-aware before asking them to complete a design task is referred to by Visser, Stappers, van der Lugt and Sanders as “sensitization” [29]. Sensitization is described as “a process where participants are triggered, encouraged and motivated to think, reflect, wonder and explore aspects of their personal context in their own time and environment” [29] (p. 5). The authors also suggested that sensitization carried out over a longer period of time helped participants to access and express their experiences, and the information learned depended on the length and depth of the sensitizing process [29].

**Figure 5:**
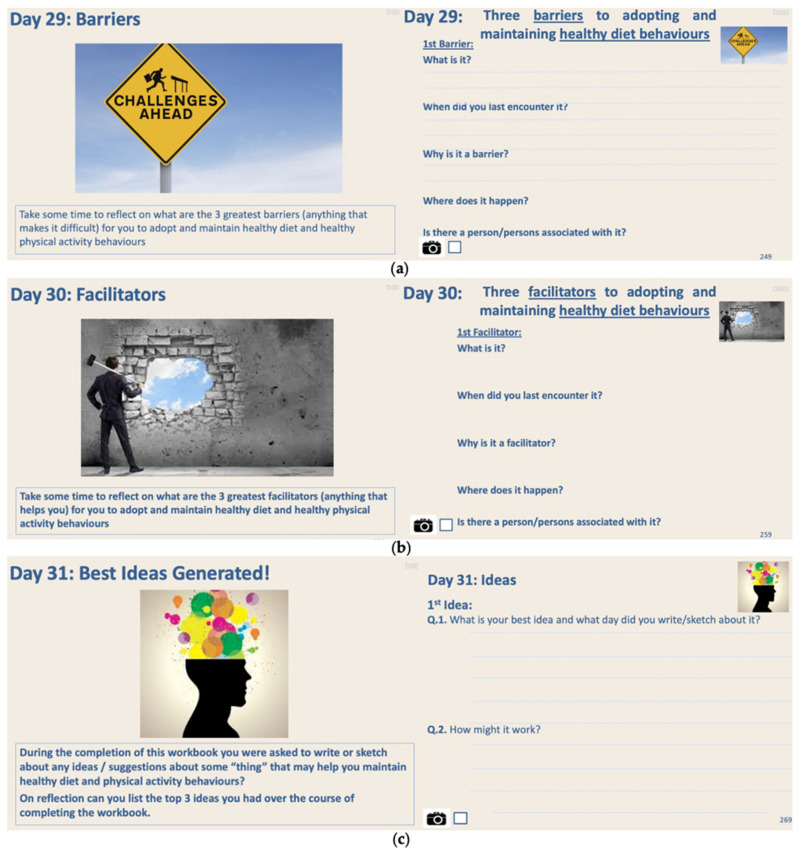
The design probe asked participants to comment on (**a**) barriers and (**b**) facilitators for diet behaviour and to provide some rationale around the selection of those barriers and facilitators. This was repeated for physical activity barriers and facilitators. Day 31 asked the participants to select their best ideas of those identified during Days 7–28 (**c**). Day 31 asked the participants to select their best ideas of those identified during Days 7–28 (**c**).

The probe finishes by asking the participants to draw on their extended period of self-reflection to answer some “design” questions, tasks (Days 29–31) (Figure 5). On Days 29 and 30, participants were asked about their barriers and facilitators to diet and physical activity behaviours and to provide some rationale around these barriers and facilitators, as we hypothesized that participants may provide greater insights at this point in the process than they would have earlier in the probe, as they were by now becoming accustomed to the process of self-reflection. From Day 7 to Day 30, we also asked participants to express their ideas about some “thing” that may help them maintain healthy diet and physical activity behaviours and for Day 31 to choose their best three ideas.

### 2.5. Data Analysis

Data analysis was only carried out on the data from Days 29, 30 and 31, where the participants were asked to describe diet and physical activity barriers and facilitators and the best of the ideas that they had identified during Days 7–28. The data from Days 1–28 were not analysed as these parts of the Design Probe were designed to sensitize the participants to topics queried on Days 29–31.

All data from Days 29–31 of the Design Probes were copied verbatim into Microsoft Word for analysis. We used the reflexive thematic analysis (TA) implemented through theoretical transparency and awareness of philosophical assumptions outlined by Braun and Clarke [30], which is a refined version of their original approach [31]. The reflexive TA approach involves six steps in data analysis:

- Familiarising yourself with the data and identifying items of potential interest
- Generating codes
- Generating initial themes
- Reviewing initial themes
- Defining and naming themes
- Producing the report [30,31]

We also followed the detailed guidelines outlined by Nowell et al. [32] in carrying out the thematic analysis, which documents a series of components within each of the aforementioned six steps as a means of establishing trustworthiness during each phase of thematic analysis.

We adopted a hybrid approach to coding using a combination of inductive and deductive analysis [33], allowing an exploration of raw data (inductive) and theoretical concepts (deductive). No a priori coding was used; however, data were clustered under the probe headings of diet barriers, diet facilitators, physical activity barriers, physical activity facilitators and best ideas to maintain healthy diet and physical activity behaviours.

Coding was carried out collaboratively as a group with three members of the research team (K.C., G.Ó.L. and L.Q.). Codes were reviewed and iterated several times, discussions were audio recorded and disagreements resolved through discussion. All context-specific data were considered; however, only barriers, facilitators and ideas were coded. Data where participants did not answer the question asked were not included for analysis. The coding was carried out at a semantic level, as objectively as possible. Each piece of data was only given one code; however, a code could be assigned to multiple themes. Patterns and connections between the codes, initial themes, final themes and sub-themes were developed in an iterative manner. Themes are presented in order of most frequently mentioned.

## 3. Results

Design Probes were sent to 21 participants with type 2 diabetes and returned fully or partially completed by 18 participants (three withdrew before the study commenced and did not provide a reason for this) with 13 Design Probes fully completed (five participants did not fully complete the Design Probe and three did not provide a reason for this). 

Data from 13 participants (Table 1) who fully completed the Design Probe were included in the qualitative analysis of days 29 (barriers), 30 (facilitators) and 31 (best ideas).

### Barriers to Healthy Diet Behaviours

Participants were asked to: identify “three barriers to adopting and maintaining healthy diet behaviours” and eight themes were created from the participants’ data:

- Food environment
- Mental health
- Work schedule
- Planning
- Social support
- Cravings
- Economic circumstances
- Energy

### Facilitators to Healthy Diet Behaviours

Participants were asked to: identify “three facilitators to adopting and maintaining healthy diet behaviours” and four themes were created, with new themes marked with a *.

- Planning
- Food environment
- Social support
- Economic circumstances

### Barriers to Healthy Physical Activity Behaviours

Participants were asked to: identify “three barriers to adopting and maintaining healthy physical activity behaviours”, nine themes were created, with new themes marked with a *.

- Physical health *
- Mental health
- Social support
- Motivation *
- Energy
- Weather *
- Work schedule
- Physical environment *
- Economic circumstances

### Facilitators to Healthy Physical Activity Behaviours

Participants were asked to: identify “three facilitators to adopting and maintaining healthy physical activity behaviours”, eight themes were created with new themes marked with a *.

- Social support
- Planning
- Mental health
- Physical health
- Motivation
- Physical environment
- Weather
- Economic circumstances

### Best Ideas 

Participants were asked to: identify “any ideas/suggestions about some “thing” that may help them maintain healthy diet and physical activity behaviours” and seven themes were created with new themes marked with a *.

- Planning
- Mental health
- Social support
- Food environment
- Education *
- Physical health
- Work schedule

The different themes will now be defined and snippets of text relating to diet and physical activity barriers and facilitators presented for these different themes.

### Food Environment

The Food Environment was defined as the environment or location where the participant may encounter, purchase, eat or prepare food. The food environment was divided into four sub-themes (shopping, home, social and work food environments). 

Shopping food environment diet barriers:

“When I shop for groceries, the ‘non-healthy’ foods are very tempting to buy…I have to make a special effort not to buy such foods”

P9 (F)

Shopping food environment diet facilitators:

“supermarket…availability of healthy foods…easier to make better decisions”

P11 (F)

Home food environment diet barriers:

“At home…I’m more likely to eat carbs in the form of bread”

P4 (F)

Home food environment diet facilitators:

“being organised & having healthy eating choices in my house”

P9 (F)

Social food environment diet barriers:

“people around me eating sweet things…I get tempted to eat/overeat this kind of food”

P9 (F)

Social food environment diet facilitators:

“In our local café…availability of healthy food”

P11 (F)

Work food environment diet barriers:

“I spend all summer on the road which leads to ‘grazing’ on the go”

P2 (M)

Work food environment diet facilitators:

“finding quick ways to bring good food to work”

P4 (F)

Food environment ideas:

“remove ‘unhealthy’ food from my house to reduce the ‘temptation’…having alternatives to snack on/make my meals from”

P9 (F)

“When on the road, don’t rely on service stations…take packed lunches”

P2 (M)

### Mental Health

The theme of Mental Health was defined in this study as cases where the participant identified negative emotions of depression, anxiety, low self-esteem, or negative self-perceptions as influencing diet and physical behaviours:

Mental health diet barriers:

“Depression, leads to complete self-neglect and loss of energy”

P6 (M)

“stress, or worry, when I feel stressed…I turn to food for comfort…I eat in secret”

P9 (F)

Mental health physical activity barriers:

“my size makes me embarrassed so I don’t go swimming which I love”

P4 (F)

Mental health physical activity facilitators:

“if house happy, I am more likely to do more exercise”

P3 (F)

Mental health ideas:

“behave and act much more positively”

P13 (M)

“Try to keep as stress free as possible”

P10 (F)

### Work Schedule

The theme of Work Schedule referred to participants’ work schedule, what time they start and finish work, whether or not their work shifts are changing. The issue of how work schedule impacts the individual’s ability to engage in healthy diet and physical activity behaviours is highlighted by this theme.

Work schedule diet barriers:

“my hours are not set…hard to plan”

P1 (M)

Work schedule physical activity barriers:

“leave home 5–6 a.m. and return 6–8 p.m.…leaves little time for any real physical activity”

P2 (M)

Work schedule ideas:

“make work fit around my life and not the other way around”

P13 (M)

### Planning

The theme of Planning refers to the role of planning in supporting healthy diet and physical activity behaviours. The diet aspect of this theme was divided into sub-themes of Work food planning, Home food planning, Food shop planning and Social food planning. Work food planning was defined as the challenges around planning healthy meals at work and planning either to purchase food at work or prepare in advance food to bring to work. Home food planning was defined as planning what food to have in the home, planning to prepare food, planning the timing and content of meals. Food shop planning was planning to purchase appropriate food. Social food planning was planning social food interactions. Physical activity planning was defined as planning physical activity including the type, frequency, intensity and duration of physical activity.

Diet planning:

Home food planning barriers:

“Not eating regularly, I tend to miss meals and then I overeat”

P3 (F)

Home food planning facilitators:

“being organised & having healthy eating choices in my house, helps me make healthy eating choices”

P9 (F)

Food shop planning facilitators:

“Planning what to eat and shopping accordingly (in advance) rather than relying on picking something up last minute”

P2 (M)

Social food planning facilitators:

“When going out to eat, choosing a venue with healthy eating choice...I can enjoy a meal out”

P9 (F)

Work food planning barriers:

“hours are not set…hard to plan”

P1 (M)

Work food planning facilitators:

“finding quick ways to bring good food to work”

P4 (F)

Physical activity planning

Physical activity planning facilitators:

“having a routine—a set time every-day to go for a walk…I don’t plan anything else for that time”

P9 (F)

Planning ideas:

“Following a plan or schedule, taking the thinking out of it, something to follow and become routine.”

P12 (F)

“Being organised & planning healthy meals in advance…by reducing / eliminating impulse eating of non-healthy foods/meals”

P9 (F)

“Try to make time for some activity every day”

P2 (M)

### Social Support

The theme of Social Support was defined as the level of support from family or friends in adopting healthy behaviours and also included the positive/negative influences, interactions or relationships with family or friends and their role in healthy behaviours. 

Social support diet barriers:

“friends, industry colleagues, social life: i.e., eating away from home…usually involve rich hotel foods”

P2 (M)

Social support diet facilitators:

“Wife, she’s always willing me to better myself”

P1 (M)

Social support physical activity barriers:

“having a ‘buddy’ to go for a walk with, I feel I would be much more likely to exercise”

P9 (F)

Social support physical activity facilitators:

“a friend helps me keep focused, keeps me motivated”

P9 (F)

Social support ideas:

“re-join my Slimming World group, will help me to maintain a healthy eating & physically active lifestyle”

P9 (F)

### Diet Cravings

Diet cravings are defined as a powerful urge or desire to eat certain foods or having difficulty resisting certain foods.

Diet craving barriers:

“When I have the munchies…I crave chocolate and crisps”

P11 (F)

### Economic Circumstances

The theme of Economic Circumstances refers to the influence of general financial circumstances on healthy diet and physical activity behaviours:

Economic circumstances diet barriers:

“fresh food…especially can be expensive”

P10 (F)

“If I do not have enough money, I tend to eat junk food”

P5 (F)

Economic circumstances diet facilitators:

“a job… provides money to be able to buy better food”

P6 (M)

Economic circumstances physical activity barriers:

“without money I am unable to pay for the gym”

P6 (M)

Economic circumstances physical activity facilitators:

“A job… forces me in another rhythm and provides money for the gym”

P6 (M)

### Energy

The theme of Energy was defined as ‘energy levels’ influencing healthy diet and physical activity behaviours.

Energy diet barriers:

“tiredness… don’t seem to have the energy after a long day at work”

P1 (M)

Energy physical activity barriers:

“If I am too tired, I do not feel like exercising”

P5 (F)

### Physical Health

Physical health was defined as the presence of a co-morbidity, injury, pain, physical limitation or inability influencing healthy diet or physical behaviours: 

Physical health physical activity barriers:

“arthritis…I am not able to walk very far without pain”

P4 (F)

Physical health physical activity facilitators:

“With the weight off, I have more energy, doing more”

P11 (F)

Physical health ideas:

“Swimming causes no pain on my feet…I’m a very good swimmer…just have to stop being embarrassed about my weight but the more weight I lose the easier that will be”

P4 (F)

### Motivation

The theme of Motivation was defined as the presence or absence of a desire to adopt and maintain healthy physical activity behaviours:

Motivation physical activity barriers:

“Motivation/commitment: I always give up, I don’t know why”

P13 (M)

Motivation physical activity facilitators:

“Signing up for charity walks…makes me get out and train”

P1 (M)

### Weather

Bad weather was defined as weather which influenced participants engaging in physical activity.

Weather physical activity barriers:

“Weather… it’s a barrier to most of the physical activities I do”

P2 (M)

Weather physical activity facilitators:

“fine weather makes me more adventurous…my physical activity is weather dependent”

P2 (M)

### Physical Environment

The physical environment was defined as the physical environment in which the participant lives, including their home and neighbourhood. 

Physical environment physical activity barriers:

“I am afraid of tripping or falling when I go for a walk”

P3 (F)

“Lack of garden…gardening brings me out of the house”

P6 (M)

Physical environment physical activity facilitators:

“Garden Allotment…gardening provides healthy exercise”

P6 (M)

### Education

The theme of education was defined as information, advice and support being provided in a written or a class format to assist in adopting and maintaining healthy diet and physical activity behaviours.

Education diet/physical ideas:

“do the 10 week course with CROI, I learnt so much from that”

P1 (M)

“re-join my Slimming World group…will help me to maintain a healthy eating & physical activity lifestyle”

P9 (F)

## 4. Discussion

The aim of this study was to design a Design Probe as a sensitization platform for type 2 diabetes participants to: reflect deeply on their emotions and lived experiences (and the context of those) and on completion of that sensitization process to document their perceived barriers and facilitators to healthy diet and physical activity behaviours. The identified barriers to healthy diet behaviour included themes of the food environment, mental health, work schedule, planning, social support, diet cravings, economic circumstances and energy. Similar barriers (apart from the food environment and motivation) were observed for physical activity in addition to physical health, weather and the physical environment. Themes identified as facilitators for healthy diet behaviour included: planning, the food environment, social support and economic circumstances. Similar facilitators were identified for healthy physical activity behaviours in addition to physical health, mental health, the physical environment and weather. 

The barriers identified in this study influencing diet behaviour are consistent with earlier qualitative research which highlighted themes of social food environment [34,35,36], mental health [34,37,38], work related [34,36,37,38], planning [39], social support [34,36,37,39,40], food cravings [34,36,41] and economic circumstances [38,39,40,41]. Other themes highlighted previously: ‘feeling deprived due to following a diet’ [34,36,37,38], ‘discouraged due to lack of results’ [34,36], ‘negative perceptions of diet’ [37,40,42], ‘lack of knowledge’ [37,38,40,41,43] and ‘lack of motivation’ [41,42] were not reported in this study. The differences in themes observed in our study compared to other similar qualitative studies may be due to the fact that participants in our study were already part of a programme compared to participants in other studies who had not completed a supervised diet and physical activity programme [34,36,41,42].

The barriers influencing healthy physical activity behaviours identified in this study were similar to those for healthy diet behaviours and are consistent with earlier research which identified similar themes of physical limitations [44,45], mental health [44,46], social support [44,47,48], ‘lack of motivation’ [35,47], fatigue or ‘lack of energy’ [41,49], the physical environment [44,45] [50], weather [44,47,48], work schedule [48] and economic circumstances [50]. Other factors such as planning or lack of time [50] [48], a lack of physician advice [48], lack of knowledge about exercising [44,45] and cultural issues [35,44,50] were not reported in this study.

Facilitators influencing healthy diet behaviours in this study are largely consistent with previous studies that also identified planning [41,51], the food environment [52], availability of social support [35,41,43] and education or providing information and knowledge translation [35,41].

Facilitators to increasing healthy physical activity behaviours identified here are similar to previous studies that included social support [35,41,45,46], planning [41,45], improving mental/physical health [45]. Other factors previously identified such as information and knowledge translation [41], goal setting [45,53], tracking [45], physical activity being enjoyable, culturally appropriate and gender-specific [35] were not reported as themes in this study.

The themes of the food environment, planning, social support and economic circumstances were reported by participants as both barriers and facilitators to adopting and maintaining healthy diet behaviours. Participants frequently highlighted the food environment, reporting how the availability of unhealthy foods and perceived expense of healthy foods in shopping, home, social or work environments was a barrier to healthy diet behaviours.

Increased availability, reduced cost of highly processed foods [2,54,55] and manipulated food taste [56] have coincided with increased intake of processed foods high in sugar, fat, salt, additives, low in fibre and low in nutrient value [57]. This environment of highly addictive, palatable and available foods, marketed [58] and engineered to be consumed in excess, is conducive to developing obesity and type 2 diabetes [57]. The importance of changing behaviour through changing the dietary environment in type 2 diabetes has been highlighted recently [11]. Possibly the first step in navigating the food environment might be increasing our awareness of it and its role in our diet behaviour. Firstly, looking at what we eat, when, where, why, how much and with whom, and planning accordingly can help this process. Action planning has been reported as a key technique in changing diet and physical activity behaviour [3,59,60].

Participants frequently reported the theme of planning as a facilitator linking it to the food environment through exercising greater control of their food environments, as one participant said: “Planning what to eat and shopping accordingly (in advance) rather than relying on picking something up last minute” P2(M). Participants suggested that a lack of planning may lead to unhealthy diet behaviours, whereas planning may make healthy diet decisions easier: “being organised & having healthy eating choices in my house, helps me make healthy eating choices” P9(F). The PRIME theory of motivation [61] proposes that five sub-systems of planning, evaluation, motivation, impulses and responses all interact and are influenced by the immediate external and internal environment [62] which suggests that the themes of planning, food environment and cravings identified in this study are all linked.

The theme of mental health was frequently reported as a barrier for healthy diet behaviours; however, mental health was not identified as a facilitator, although it was frequently mentioned in participants’ ideas. Several participants reported negative emotions such as depression, anxiety, stress as a barrier to adopting and maintaining healthy diet behaviours. Participants identified coping strategies such as engaging social support of family and friends and enhancing self-esteem as ideas to improve mental health. Mental health in type 2 diabetes is affected by comorbidities [63], challenges of managing physical activity, diet and medication [64], with diabetes distress [63,65,66,67], anxiety [68,69,70] and depression [63,68,71,72] prevalent. However, interventions creating positive cognitions, feelings or behaviours reduce depressive symptoms, enhance immune system [73] and significantly enhance well-being [74]. Experiencing the present moment through Mindfulness can help us manage the adverse effects of stress [75,76,77], anxiety and depression [76,78,79]. Practicing Mindfulness can have a positive effect on what and how we eat and drink, [80,81,82], how we move [83] and in managing type 2 diabetes [84].

Participants reported how the theme of a lack of support from friends and family can be a barrier, whereas positive influences or interactions facilitate the adoption or maintenance of healthy diet behaviours. Actively engaging support of family, friends or experts can also support participants in changing diet behaviour [35,41,43]. A recent review reported that social support was present in more than 90% of effective technology driven type 2 diabetes prevention interventions [85]. Another review identified supportive actions of communication and family collaboration, while nagging, irritation and refusing to share the burden of diabetes were classified as non-supportive interactions of family in managing diet behaviours, medication and glucose monitoring [86]. This review also highlighted the importance of including both patient and family in treatment as the majority of diabetes self-management occurs within a family environment [86]. 

The themes of physical health, mental health, social support, motivation, weather, physical environment and economic circumstances were reported by participants as both barriers and facilitators to adopting and maintaining healthy physical activity behaviours.

The theme of physical health was frequently mentioned by participants as a barrier to adopting and maintaining healthy physical activity behaviours. Many participants reported pain, physical limitations and challenges of dealing with co-morbidities of diabetes. Managing co-morbidities, additional weight [2,44,53] and pain [41,46,48,87,88] have been identified as key factors in increasing physical activity in a type 2 diabetes population, but place huge demands on participants’ energy levels and their ability to manage and control their diabetes. According to the strength model of self-control, exerting self-control in one task may deplete subsequent self-control attempts, and self-control is not an infinite pool [89,90]. Depletion of self-regulatory resources has been shown to reduce participation in exercise [91]. Bandura observed that amongst conditions requiring continuous self-management, “none is more demanding than diabetes” in co-ordinating self-care activities such as diet and exercise behaviours [92] (p. 288).

The theme of mental health was frequently reported as a barrier to physical activity but less frequently as a facilitator. Participants reported how experiencing negative emotions such as depression, anxiety, stress and embarrassment about weight were barriers to adopting and maintaining healthy physical activity behaviours. Some participants identified positive mental health associated with increased physical activity through the availability of social support and better weather. Exercise is associated with enhanced mental health and improved condition in type 2 diabetes [93]. A large recent study from the USA reported that physical exercise was associated with reduced mental health burden; however, more exercise was not always associated with better mental health and certain exercise types, durations and frequencies were better than others [94]. 

Social support was the most frequently mentioned facilitator for adopting and maintaining healthy physical activity. Engagement of support of family or friends has been associated with increased physical activity levels in type 2 diabetes interventions [8,10,60], as social support and modelling of active lifestyles by friends and families contribute to perceived self-regulatory efficacy to keep physically active [95] and “success in regular exercise is heavily dependent on self-regulatory capacity” [92]. Adopting and maintaining physical activity requires the development of self-regulatory capabilities such as goal setting, self-monitoring and enlisting supportive feedback [92]. The theme of planning and prioritizing time for physical activity, identified in this study, is also very important, as Bandura suggests that “without some explicit time management, exercise readily falls victim to competing activities” [92] (p. 415). 

The themes of a ‘lack of motivation’ and ‘lack of energy’ were frequently mentioned as barriers to adopting and engaging in healthy physical activity. Motivation is a complex construct regarding physical activity with some participants reporting perceived lack of motivation and lack of energy as barriers to physical activity. Different authors have different perceptions and definitions of motivation; however, in participants with type 2 diabetes reduced motivation and low energy levels may stem from the reduced self-regulatory capacity associated with coping with type 2 diabetes and reduced self-efficacy towards physical activity. Self-efficacy is significantly correlated with physical activity [5,96]; however, self-inefficacious thinking can hamper future efforts to increase physical activity [92]. According to Bandura, “perceived efficacy affects every phase of habit change in physical activity as it does in eating habits” [92] (p. 353).

Moving from a sedentary to a more active lifestyle involves challenges and setbacks; therefore, individuals must be able to identify barriers to regular physical activity and programmes need to include strategies for relapse prevention [3,10]. The conclusion of a recent review that “any physical activity is better than none” [97] is probably an appropriate starting point for sedentary individuals as programmes designed to make regular exercise a habit should include incentives, start at moderate levels of exertion and gradually increase the intensity, frequency and duration [92].

### 4.1. Design Ideas

**Figure 6:**
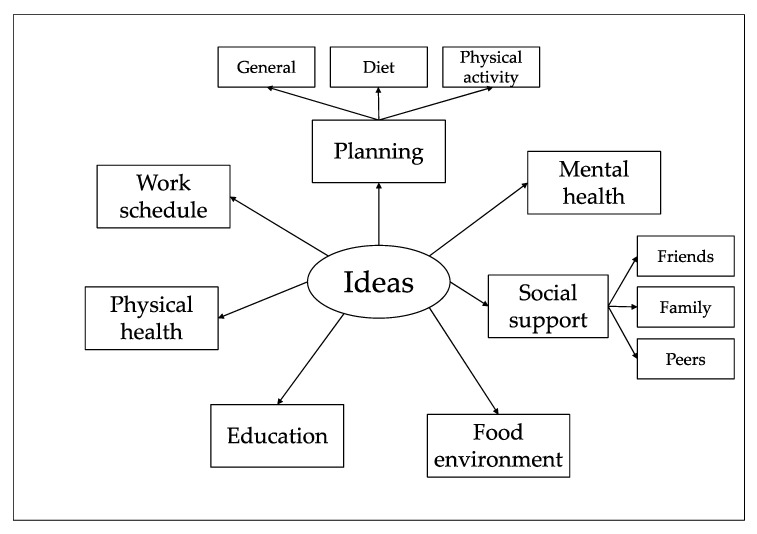
Ideas thematic map.

Ideas generated by participants about changing diet or physical activity behaviour revolved around the themes of planning, mental health, social support, and creating a healthier food environment, education, physical health and work schedule (Figure 6). Many of the ideas identified in this study have been associated with successful outcome changes in diet and or physical activity interventions in type 2 diabetes or obese adults such as planning [3,59,60], social support [8,10,60], improving mental health [98] and creating a healthier food environment [11]. It is planned that ideas identified will be used to inform the design of a future behaviour change artefact.

### 4.2. Empathy for Design

In addition to its role in ‘sensitization’, making the participants more self-aware before asking them to complete a design task has been referred to [29], the Design Probe exercise also provided the research team with deep insights into the lived experiences of persons with T2D and the context in which those experiences occur.

These insights have helped the team to develop greater empathy with persons with T2D, as the team embarks on the next phase of this research project: artefact design for diet and physical activity behaviour change in persons with T2D. It is hoped that this developed empathy will, as Kouprie et al. have suggested, inform and inspire the design team to create products that fit the user’s needs [14].

### 4.3. Strengths and Limitations 

Extensive pilot testing was carried out as part of the iterative development of the Design Probe. A significant level of commitment was required by the participants in that they had to engage with the Design Probe each day for 31 days, spending between 10–15 min each day with probe tasks. Considering the level of commitment required, the response rate (fully completed probes) of 72% was a very positive outcome. Our multidisciplinary team approach incorporating behaviour change, design, engineering and medical expertise was a strength of the study. Design Probes were completed for 31 days which allowed participants a significant period of self-reflection (28 days) on their lived experience.

The self-report nature of this method may be a limiting factor as some participants struggled to identify barriers and facilitators. This method is also limited by participant engagement as a sub-optimal engagement by a participant renders the resulting data of limited value. There may be bias in the sample as participants who volunteered to participate were already highly motivated and may not be a true representation of this population in addition to the small sample (*n* = 13) who fully completed the Design Probes.

### 4.4. Future Directions 

The asynchronous benefits of Design Probes, allowing participants to reflect on and complete tasks on their own time, may potentially be applied to other sensitive or complex behaviours. Testing the efficacy of the role of self-reflection in initiating behaviour change will further our understanding of behaviour change. On reflection, using the Design Probe purely as a sensitization tool for 28 days and then following up quickly afterwards by interviewing the participants using a semi-structured interview to identify diet and physical activity barriers, facilitators and ideas may have been a better approach in helping participants explore their barriers and facilitators in greater depth.

## 5. Conclusions

Our Design Probe yielded valuable data on the lived experiences of persons with T2D, their perceived barriers and facilitators to diet and physical activity behaviour change and their design ideas around diet and physical activity behaviour change. This study reported themes of the food environment, planning, social support, mental health, work schedule, cravings, energy and economic circumstances linked to changing diet behaviour. Similar themes were created for physical activity with the additional themes of physical health, weather, motivation and the physical environment reported.

## Figures and Tables

**Table 1:** Participant data (*n* = 18).

#	Sex	Age (Range/Years)	T2D Duration (Years)	Probe Status	Reasons for Incomplete Probe	Education Highest Level	Race/Ethnicity
1	M	35–44	1 to 2	Fully Completed	NA	2nd Level	White/Irish
2	M	55–64	6 to 10	Fully Completed	NA	3rd Level (L6)	White/British
3	F	55–64	>10	Fully Completed	NA	3rd Level (L5)	White/English
4	F	55–64	6 to 10	Fully Completed	NA	3rd Level (L8)	White/Irish
5	F	35–44	1 to 2	Fully Completed	NA	3rd Level (L5)	White/Irish
6	M	45–54	>10	Fully Completed	NA	2nd Level	White/Dutch
7	M	35–44	6 to 10	Fully Completed	NA	2nd Level	White/Irish
8	F	55–64	4 to 6	Fully Completed	NA	3rd Level (L8)	White/Irish
9	F	55–64	>10	Fully Completed	NA	3rd Level (L6)	White/Irish
10	F	55–64	2 to 4	Fully Completed	NA	3rd Level (L6)	White/Irish
11	F	55–64	4 to 6	Fully Completed	NA	3rd Level (L7)	White/Irish
12	F	55–64	>10	Fully Completed	NA	2nd Level	White/Irish
13	M	25–34	1 to 2	Fully Completed	NA	3rd Level (L8)	White/Irish
14	M	>65	>10	(11 days)	No reason provided	2nd Level	White/Irish
15	F	NR	NR	(8 days)	In hospital	NR	White/Irish
16	F	55–64	2 to 4	(12 days)	No reason provided	Primary level	White/Irish
17	M	NR	NR	(15 days)	Family member in hospital	NR	White/Irish
18	F	NR	NR	(9 days)	No reason provided	NR	White/Irish

## Data Availability

Not applicable.
